# Case Report: A Case of Perifoveal Exudative Vascular Anomalous Complex With a Good Prognosis

**DOI:** 10.3389/fmed.2021.757313

**Published:** 2021-12-13

**Authors:** Min Fu, Pan Hu, Gang Zhang, Ludonghan Huang, Huan Xu, Ju Huang, Meihui Wu, Yanli Chen

**Affiliations:** ^1^Department of Ophthalmology, Daping Hospital of Army Characteristic Medical Center, Chongqing, China; ^2^Department of Medicine, 953 Hospital of People's Liberation Army (PLA), Xizang, China; ^3^Nursing Teaching and Research Section, Daping Hospital of Army Characteristic Medical Center, Chongqing, China

**Keywords:** perifoveal exudative vascular anomalous complex (PEVAC), micropulse laser (MPL), age-related macular degeneration (AMD), case report, good prognosis

## Abstract

**Significance:** Perifoveal exudative vascular anomalous complex (PEVAC) is a unique clinical lesion. It manifests as isolated lesions and is easily misdiagnosed. Thus far, few PEVAC case reports have been published. PEVAC is typically inconsistent with other reported macular lesions.

**Purpose:** To report our 24-month follow-up experience on the treatment of PEVAC with a micropulse laser (MPL).

**Case Report:** A 56-year-old Chinese woman with no history of other diseases complained of decreased vision in her left eye that had persisted for more than 1 year. Comprehensive ophthalmic examinations were performed, including a vision test, slit lamp fundus exam, optical coherence tomography (OCT), optical coherence tomography angiography (OCT-A), fluorescein fundus angiography (FFA) and indocyanine green angiography (ICGA). Intravitreal injection of ranibizumab was ineffective, and bleeding, exudation and visual acuity were not improved. After two rounds of micropulse laser (MPL) treatment, the patient was followed up, and the prognosis was good.

**Conclusion:** PEVAC is very rare, and early diagnosis is important, as the lesions readily cause irreversible damage. Our results indicate that an MPL can be used as an alternative treatment for PEVAC patients.

## Introduction

Perifoveal exudative vascular anomalous complex (PEVAC) is a unique clinical lesion. It manifests as isolated lesions in close proximity to the macula; thus, it is easily misdiagnosed. PEVAC is an elusive, rare lesion that has mainly been reported in case studies and is usually non-responsive to anti-vascular endothelial growth factor (VEGF) treatment. In the absence of capillary ischemia or inflammation, the pathogenesis of PEVAC may be progressive degeneration of retinal endothelial cells, which may also explain the ineffectiveness of anti-VEGF treatment (1, 2). In summary, PEVAC is an isolated perifoveal vascular lesion. The characteristics of PEVAC patients according to optical coherence tomography (OCT) are consistent in existing reports. Patients with PEVAC may have diabetes or hypertension; additionally, PEVAC can occur in healthy individuals or in those with myopic macular degeneration and age-related macular degeneration (AMD). It may also be related to type 3 neovascularization (T3NV)/retinal angiomatous proliferation (RAP), which is inconsistent with the clinical features of macular telangiectasia type 1 (MacTel 1) and RAP. Therefore, clinicians should be aware of the possibility that patients may have this special perifoveal disease and should focus on its relatively poor prognosis and potential prolonged recovery time.

## Case Report

**Figure 1:**
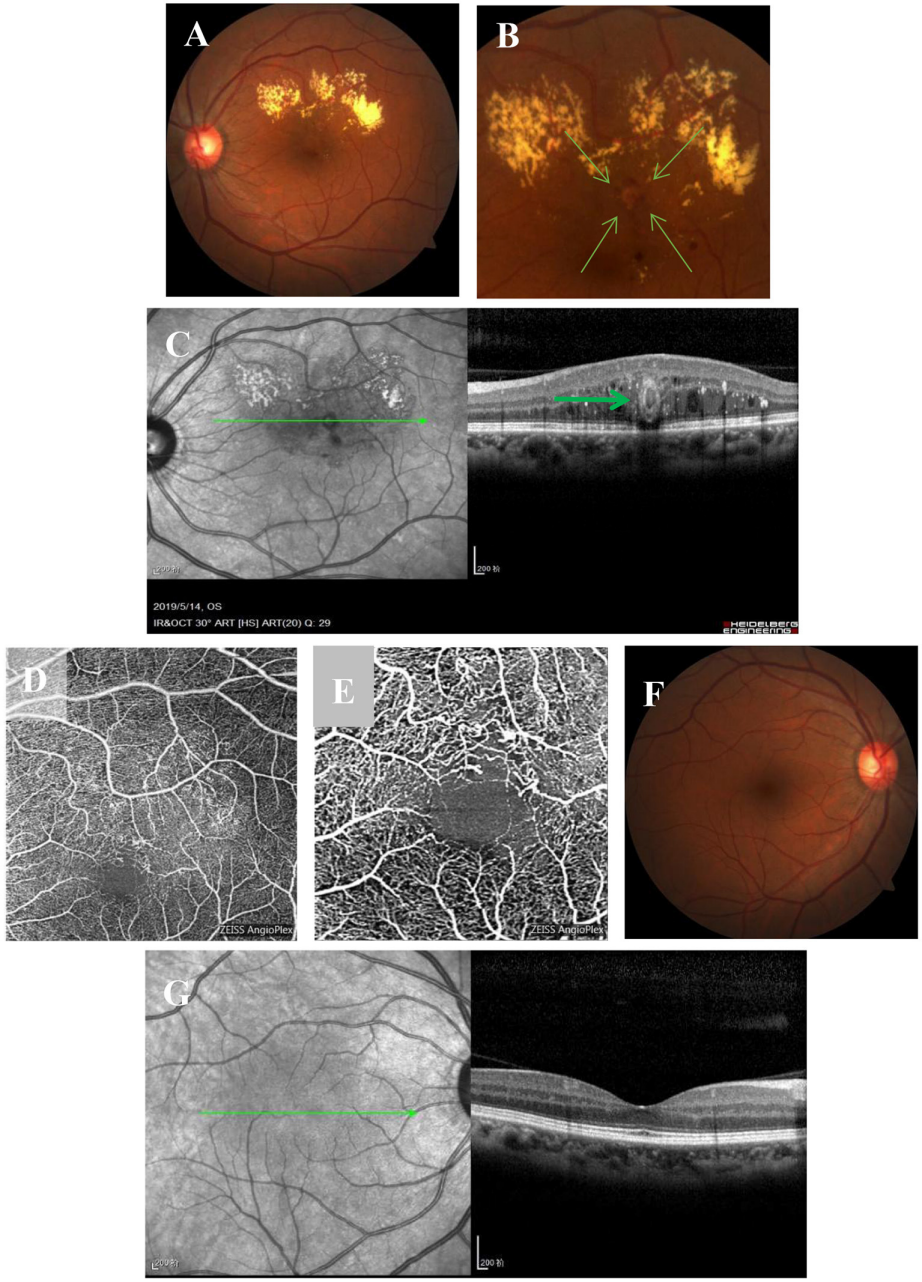
Multimodal imaging at the initial visit. **(A)** Fundus examination of the left eye shows an isolated off-white lesion near the macular area and a large level of muddy-like exudate above the macular area. **(B)** An enlarged image of the lesion. **(C)** OCT imaging shows off-white lesions on transverse scanning; the lesions are bright on the outside and black on the inside with an oval ring reflection (bold green arrow), strong point reflection between layers, and edema between layers. **(D)** OCT-A shows sparse blood vessels, superficial vascular dilatation, and a tangled nodule beside the vessels. **(E)** Enlarged OCT-A image. **(F,G)** The fundus and OCT of the right eye are normal.

A 56-year-old Chinese woman without diabetes and other diseases presented with visual acuity deterioration in her left eye for over 1 year. Ophthalmic examination revealed that her right eye vision was 20/20, her left eye vision was 20/160, and the anterior segment of both eyes was normal. However, fundus examination of the left eye showed an isolated off-white lesion near the macular area and a large amount of muddy-like exudates above the macular area (Figure 1A). An enlarged image of the lesion (Figure 1B). OCT images showed off-white lesions in the cross-sectional scan that were bright around the edges and black on the inside, with an oval circular reflex, a strong point-like reflex and edema between the layers (Figure 1C). An OCT-A scan showed sparse blood vessels with dilated superficial blood vessels and an adjacent tangled nodule (Figure 1D). Enlarged OCT-A image (Figure 1E). The fundus and OCT of the right eye were normal (Figures 1F,G).

**Figure 2:**
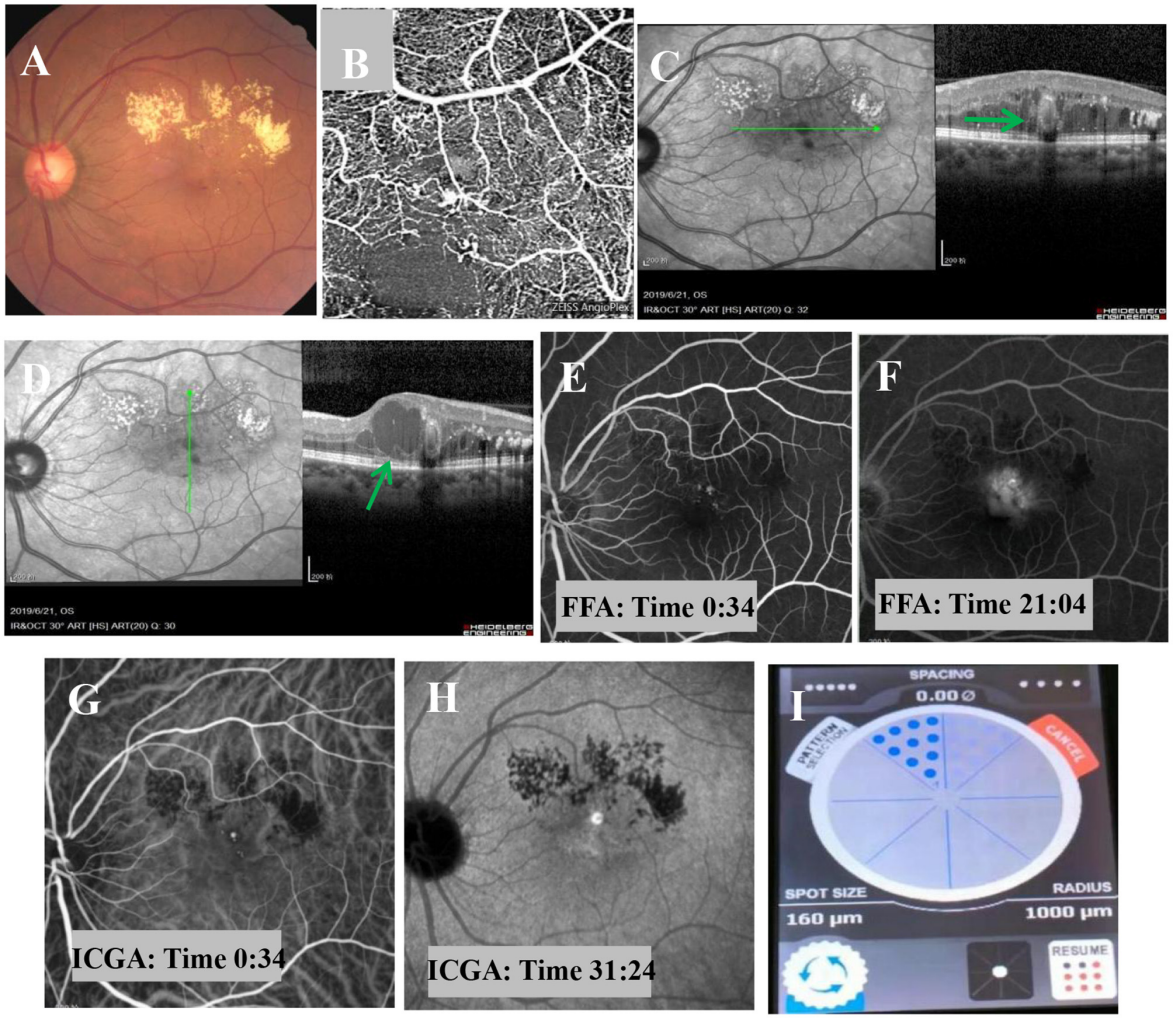
Fundus examination one month after intravitreal injection of anti-VEGF. **(A)** Fundus examination shows muddy-like exudation above the macular area, punctate hemorrhage, and no obvious change in the off-white lesions adjacent to the macula. **(B)** OCT-A shows sparse blood vessels, dilated superficial blood vessels, and a tangled nodule beside the blood vessels. **(C,D)** OCT imaging shows macular edema, dot-like hyperreflexia between layers, light on the outside and black on the inside, with no obvious change in oval structure (bold green arrow), and cystic edema in some parts of the macular area (bold green arrow). **(E,F)** FFA shows a strong fluorescent focus with a clear boundary near the macula in the early stage and obvious leakage in the later stage. **(G,H)** ICGA shows well-defined, strong fluorescence near the macula in the early stage, but no obvious leakage in the later stage. **(I)** The MPL treatment was delivered in a fan shape, covering the focus and the upper edema area.

Based on these prominent clinical features, the patient was diagnosed with PEVAC. Therefore, an intravitreal injection of anti-VEGF treatment was administered. One month after the injection, the following findings were observed, as shown in Figures 2A–H. Bleeding and focal exudation were still present, and the patient's visual acuity and focality were not improved.

**Figure 3:**
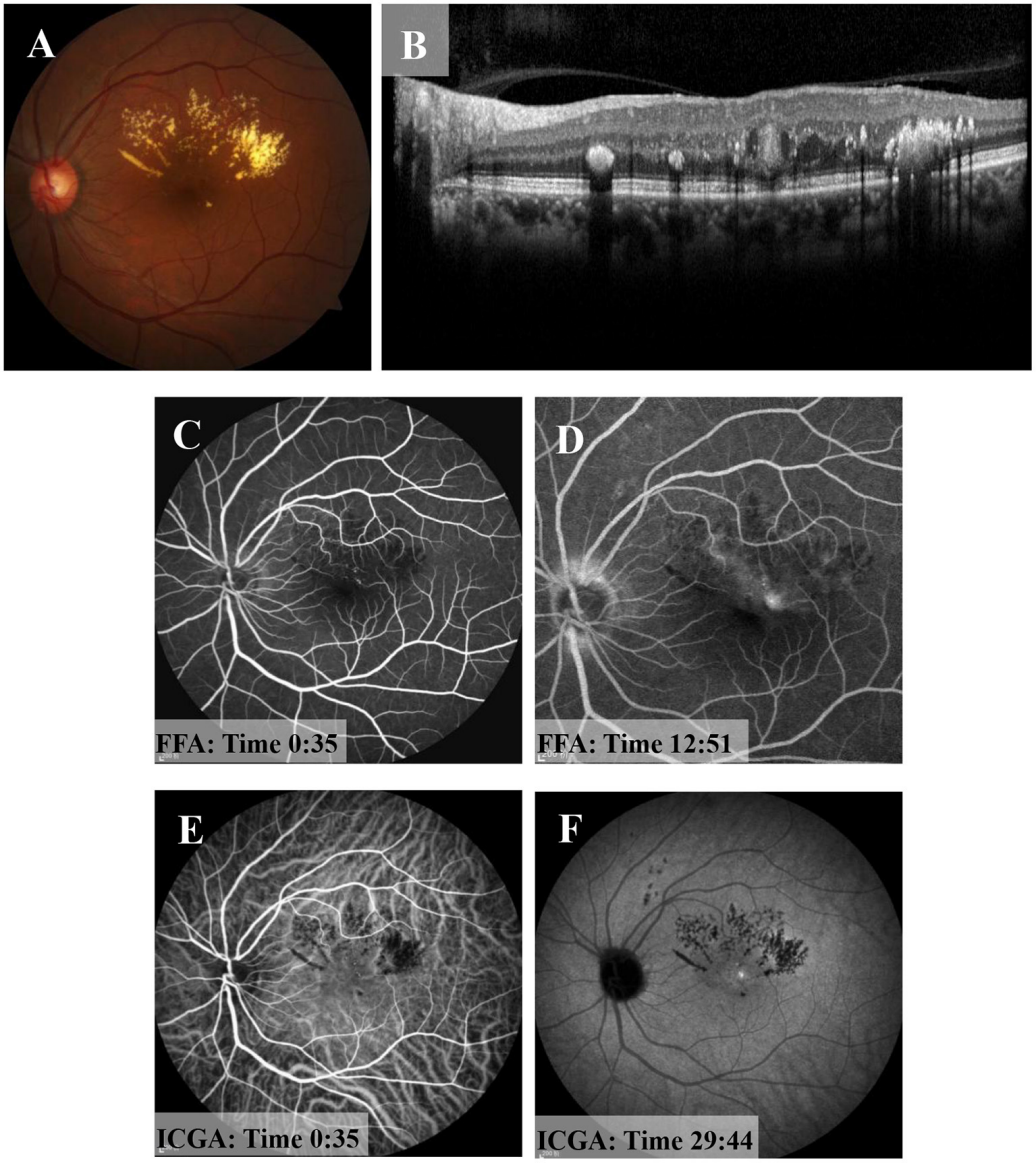
One month after the second MPL treatment. **(A)** Fundus examination of the left eye shows muddy-like exudation above the macular area and that the off-white lesions had shrunken. **(B)** OCT shows that the oval structure in macular area had become more solid and that the cavity structure had disappeared. **(C,D)** FFA shows strong fluorescent light spots near the macular area in the early stage and slight leakage in the later stage. **(E,F)** ICGA shows a light spot near macular area in the early stage, and there was no leakage in the later stage.

**Figure 4:**
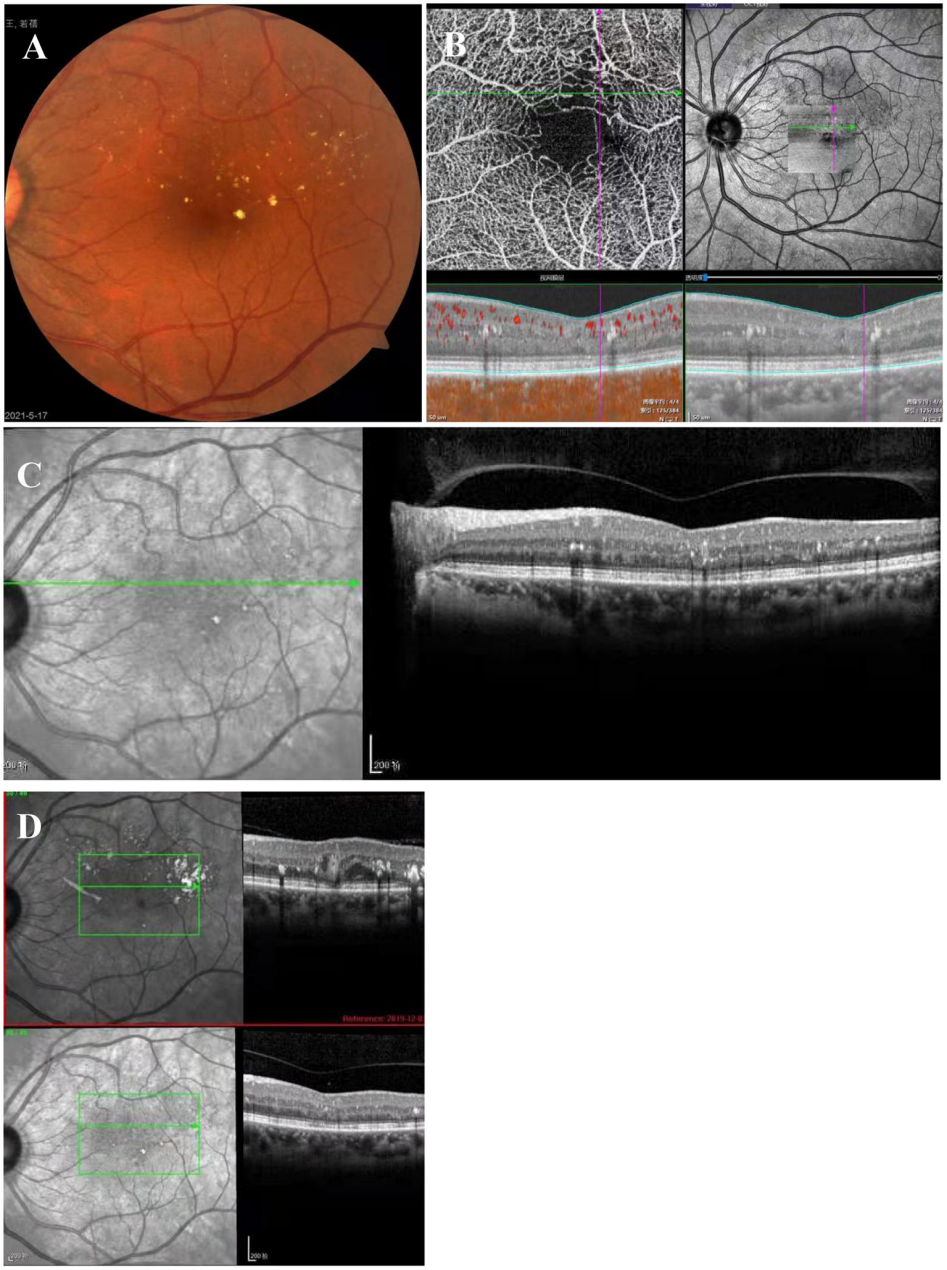
Twenty-four months after MPL treatment. **(A)** Exudates are scattered above the macular area. **(B)** OCT-A shows sparse vessels in the upper arch ring and that the tumor-like dilatation had disappeared. **(C)** OCT shows that the tumor-like structure had disappeared, and there are a few punctate hyperreflections between the layers. **(D)** In the OCT comparison chart, the tumor-like structure had disappeared.

The suggestion of PDT treatment was refused because of the high price and lack of guaranteed efficacy. Instead, MPL treatment (QUANTEL MEDICAL) was chosen, the effect of which is uncertain, after communicating with the patient, who signed the informed consent form and subsequently received a total of 2 MPL treatments. The MPL treatment was delivered in a fan shape, covering the focus and the upper edema area. The parameters were as follows, 577 nm, 160 μm, 300 mw, 0.2 s, duty cycle 5% and spacing 0 (Figure 2I). The patient underwent two MPL treatments one month apart. One month after the second MPL treatment, examination of the left eye showed that the exudation and edema were significantly improved (Figures 3A–F). After 24 months of follow-up, the prognosis of the patient was good, and the visual acuity recovered to 20/50. Examination of the left eye showed that the exudation and edema were significantly improved and that the lesions had disappeared (Figures 4A–D).

## Discussion

In 2011, Querques et al. (1) reported PEVAC for the first time. An increasing number of researchers have begun investigating PEVAC, although its pathogenesis remains unclear (1–6). In 2017, studies across 15 centers (2) showed that PEVAC is an isolated, perifoveal, angiomatous lesion that generally occurs in healthy patients but can also be accompanied by AMD and myopic macular degeneration. PEVAC has a slight or poor response to anti-VEGF treatments. The possible reasons are as follows (1): first, under an absence of capillary ischemia or inflammation, progressive retinal endothelial cell degeneration may trigger the vasogenic cellular mechanism for PEVAC; and second, the disease may be related to T3NV /RAP. In 2019, Venkatsh et al. (4) and Zhengwei Zhang et al. (6) reported the case of a patient with diabetes with PEVAC in whom anti-VEGF injection treatment was ineffective, which further indicated that PEVAC may not only occur in healthy eyes.

PEVAC should be distinguished from other periretinal vascular abnormalities. In a recent study, Mrejen-Uretsky et al. (3) reported that PEVAC may be misdiagnosed as other diseases, such as MacTel 1 and RAP. Attention should be given by clinicians to the possibility of PEVAC in the differential diagnosis. However, through multimodal imaging analysis, the characteristic imaging findings of PEVAC are obvious. For example, OCT shows that the lesion has a strong reflective ring wall and internal dark cavity, and OCT-A shows blood flow in the lesion.

RAP (T3NV) (7–10) is a special type of neovascular age-related macular degeneration (nARMD) that usually presents as isolated perifoveal vascular lesions. Stage 1 RAP is usually accompanied by small retinal edema without obvious hard exudation. PEVAC is easily confused with stage 1 RAP (11–13), but their morphologies are different on OCT. PEVAC appears as a circle-like lesion with a highly reflective signal wall and a cavity that contains a substance with uncertain reflective signals. In contrast, stage 1 RAP manifests as a hyperreflective focus without a hyperreflective wall. In addition, RAP is considered to have a poor prognosis. Anti-VEGF drugs are the first choice of therapy for nARMD; many studies have confirmed their effectiveness for the treatment of RAP (11–13), but intravitreal injection of anti-VEGF for PEVAC has no obvious effect.

Retinal arterial macroaneurysms (RAMs) are pathological dilations of the retinal artery, and most occur in elderly women over 60 years old. RAMs are associated with hypertension, diabetes, heart disease and other systemic conditions. These macroaneurys have variable clinical manifestations and are easily misdiagnosed as exudative AMD, diabetic retinopathy (DR), branch retinal vein occlusion (BRVO), telangiectasia and other hemorrhagic diseases with similar manifestations (14, 15). Unlike PEVAC, large aneurysms of the retinal artery are usually saccular or fusiform dilations of the large artery at the first three bifurcations of the central retinal artery, relatively far from the fovea (16). Most patients with retinal aneurysms have good vision after treatment (14). This is contrary to the poor vision prognosis after treatment in most patients with PEVAC (1–3).

Macular telangiectasia type 1 (MacTel 1) usually occurs in young patients and is characterized by multiple telangiectasias and microangiomas involving the temporal side of the macula. Furthermore, it is often accompanied by macular edema and hard exudation. Intravitreal injection of anti-VEGF treatment is effective (7). PEVAC exhibits perifoveal, large isolated aneurysmal changes, and two lesions have been reported in rare cases (2, 6). The common imaging feature of these PEVAC and MacTel 1 lesions is a sparse capillary network around the lesion (2, 3, 17). The relationship between PEVAC and MacTel 1 needs to be further investigated.

MPL is a repeatable, inexpensive, and minimally damaging treatment (18). The main mechanism of MPL is direct blockage of leaking microangiomas and moderate destruction of the decompensated retinal pigment epithelium (RPE) by the generation of photocoagulation. The surrounding RPE cells are then activated and proliferated, and then the function of the blood-retinal barrier is repaired and reconstructed. MPL treatment may reduce the local inflammatory response by deactivating retinal microglia. Light induces an effect on the optic nerve omentum, which causes a series of cytokine changes (19, 20).

In conclusion, according to our findings, our patient's bleeding and exudation were not alleviated by the first injection of anti-VEGF, and no improvement in vision or lesions was observed. After communication with the patient, subsequent treatment with MPL was initiated, and a beneficial treatment effect was obtained. The selective RPE injury mechanism of MPL may not be the main factor for PEVAC treatment. Instead, the photoinduced biological effect on neuroretinal tissues and cells may be responsible for improving the clinical effect of MPL in PEVAC treatment. More samples should be obtained in future in-depth research. Since PEVAC is extremely rare, retinal vein occlusion (RVO), RAP, DR and other microaneurysm-like lesions could be selected to observe the therapeutic effect and increase the applicability of MPL in further research.

## Conclusion

PEVAC appeared as a single aneurysm with exudation in this patient, and intravitreal injection of anti-VEGF was ineffective. Subsequently, two MPL treatments were administered, and a sustained positive functional result was obtained. Therefore, the pathogenesis of PEVAC and its effective treatment with MPL are worthy of further study. Here, we presented our experience with MPL to provide valuable information about this treatment to clinicians.

## Data Availability Statement

The original contributions presented in the study are included in the article/supplementary materials, further inquiries can be directed to the corresponding author/s.

## Ethics Statement

The studies involving human participants were reviewed and approved by Ethics Committee, Daping Hospital of Army Characteristic Medical Center, Chongqing, China. The patients/participants provided their written informed consent to participate in this study. Written informed consent was obtained from the individual(s) for the publication of any potentially identifiable images or data included in this article.

## Author Contributions

The data were analyzed and the manuscript was drafted by MF and PH. The manuscript was revised by YC. The results were analyzed and conclusions were made by MF, PH, and YC. Data were collected by LH, HX, and JH. The data were analyzed and integrated by GZ and MW. The final manuscript was read and approved by all authors.

## Conflict of Interest

The authors declare that the research was conducted in the absence of any commercial or financial relationships that could be construed as a potential conflict of interest.

## Publisher's Note

All claims expressed in this article are solely those of the authors and do not necessarily represent those of their affiliated organizations, or those of the publisher, the editors and the reviewers. Any product that may be evaluated in this article, or claim that may be made by its manufacturer, is not guaranteed or endorsed by the publisher.
